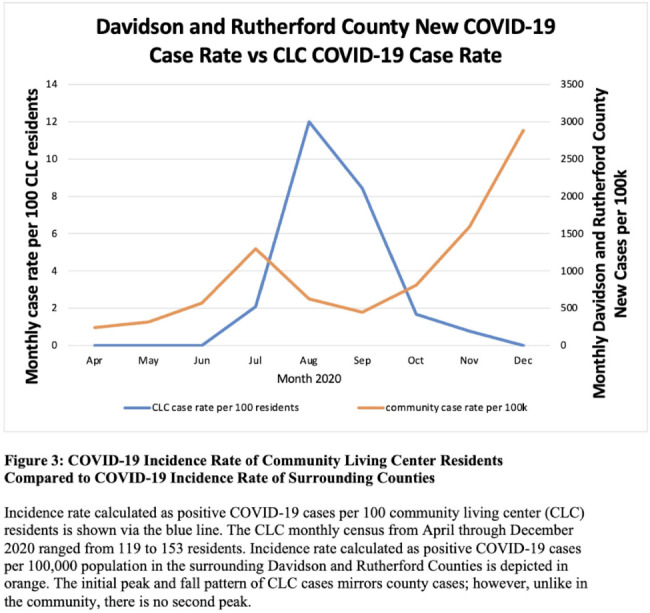# Long-term care facility employee infection prevention adherence and prevention of COVID-19 outbreaks in a high-incidence area

**DOI:** 10.1017/ash.2022.153

**Published:** 2022-05-16

**Authors:** Jennifer Cihlar, Karen Volpe, Morgan Johnson, Claudio Mosse, Christianne Roumie, Todd Hulgan, Milner Staub

## Abstract

**Background:** Long-term care facility (LTCF) employees pose potential risk for COVID-19 outbreaks. Association between employee infection prevention (IP) adherence with facility COVID-19 outbreaks remains a knowledge gap. **Methods:** From April through December 2020, prior to COVID-19 vaccination, we tested asymptomatic Veterans’ Affairs (VA) community living center (CLC) residents twice weekly and employees monthly, which increased to weekly with known exposure, for SARS-CoV-2 via nasopharyngeal PCR. Employees voluntarily completed multiple choice questionnaires assessing self-reported IP adherence at and outside work. Surveys were longitudinally administered in April, June, July, and October 2020. Changes in paired employee responses for each period were analyzed using the McNemar test. We obtained COVID-19 community rates from surrounding Davidson and Rutherford counties from the Tennessee Department of Health public data set. CLC resident COVID-19 cases were obtained from VA IP data. Incidence rate and number of positive tests were calculated. **Results:** Between April and December 2020, 444 employees completed at least 1 survey; 177 completed surveys in both April and June, 179 completed surveys in both June and July, and 140 completed surveys in both July and October (Fig. 1). Across periods, employee surveys demonstrated an increase in masking at work and outside work between April and June (63% to 95% [*P* < .01] and 36% to 63% [*P* < .01], respectively), and June to July (95% to 99% [*P* < .05] and 71% to 84% [*P* < .01], respectively) that were both maintained between July and October (Fig. 2). Distancing at work and limiting social contacts outside work significantly decreased from April to June but increased in subsequent periods, although not significantly. COVID-19 community incidence peaked in July and again in December, but CLC resident COVID-19 cases peaked in August, declined, and remained low through December (Fig. 3). **Discussion:** Wearing a mask at work, which was mandatory, increased, and voluntary employee masking outside work also increased. CLC COVID-19 cases mirrored community increases in July and August; however, community cases increased again later in 2020 while CLC cases remained low. Employees reporting distancing at work and limiting social contacts outside work decreased preceding the initial rise in CLC cases but increased and remained high after July. **Conclusions:** These data from the pre–COVID-19 vaccination era suggest that widespread, increased support for and emphasis on LTCF IP adherence, especially masking, may have effectively prevented COVID-19 outbreaks in the vulnerable LTCF population.

**Funding:** None

**Disclosures:** None

**Figure:**
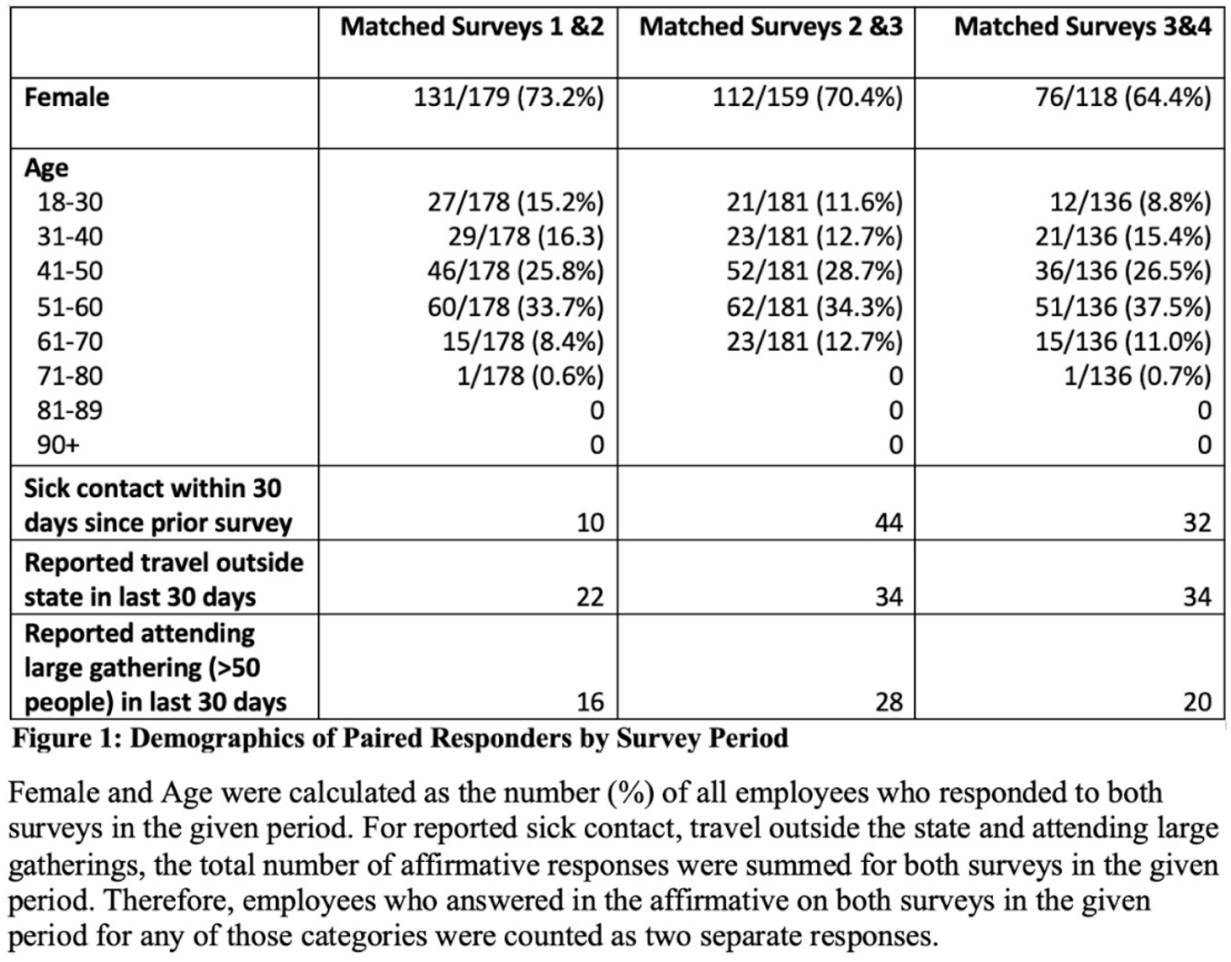

**Figure:**
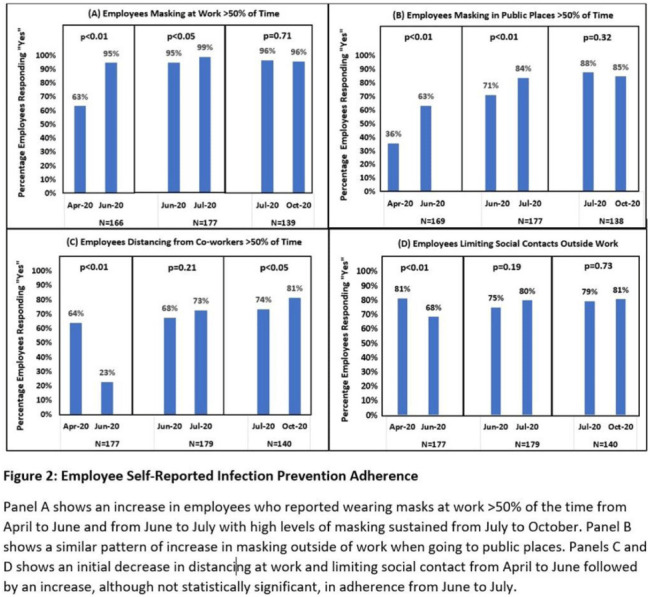

**Figure:**